# Interactive molecular causal networks of hypertension using a fast machine learning algorithm MRdualPC

**DOI:** 10.1186/s12874-024-02229-y

**Published:** 2024-08-02

**Authors:** Jack Kelly, Xiaoguang Xu, James M. Eales, Bernard Keavney, Carlo Berzuini, Maciej Tomaszewski, Hui Guo

**Affiliations:** 1 Centre for Biostatistics, School of Health Sciences, Faculty of Medicine, Biology and Health, University of Manchester, Manchester, UK; 2 Division of Cardiovascular Sciences, Faculty of Medicine, Biology and Health, University of Manchester, Manchester, UK; 3 Division of Cardiology and Manchester Academic Health Science Centre, Manchester University NHS Foundation Trust, Manchester, UK; 4 Manchester Heart Centre and Manchester Academic Health Science Centre, Manchester University NHS Foundation Trust, Manchester, UK

**Keywords:** Molecular networks, Causal inference, Machine learning, Hypertension, MRdualPC, Multi-omics integration

## Abstract

**Background:**

Understanding the complex interactions between genes and their causal effects on diseases is crucial for developing targeted treatments and gaining insight into biological mechanisms. However, the analysis of molecular networks, especially in the context of high-dimensional data, presents significant challenges.

**Methods:**

This study introduces MRdualPC, a computationally tractable algorithm based on the MRPC approach, to infer large-scale causal molecular networks. We apply MRdualPC to investigate the upstream causal transcriptomics influencing hypertension using a comprehensive dataset of kidney genome and transcriptome data.

**Results:**

Our algorithm proves to be 100 times faster than MRPC on average in identifying transcriptomics drivers of hypertension. Through clustering, we identify 63 modules with causal driver genes, including 17 modules with extensive causal networks. Notably, we find that genes within one of the causal networks are associated with the electron transport chain and oxidative phosphorylation, previously linked to hypertension. Moreover, the identified causal ancestor genes show an over-representation of blood pressure-related genes.

**Conclusions:**

MRdualPC has the potential for broader applications beyond gene expression data, including multi-omics integration. While there are limitations, such as the need for clustering in large gene expression datasets, our study represents a significant advancement in building causal molecular networks, offering researchers a valuable tool for analyzing big data and investigating complex diseases.

## Introduction

Molecular networks are important to gain insight into biological processes beyond the analysis of a single gene or molecule [1], because they help understand the interactive effects of genes on diseases. These networks have become increasingly complex as large-scale high dimensional data become prevalent. Elucidating biologically relevant information from the data is crucial but difficult. In particular, it is challenging to understand the causal role of genes and their interactions in diseases for the development of well-targeted treatments.

Recent research on causal inference using genetic information has largely focused on Mendelian randomization (MR) and Bayesian network (BN) [2]. MR uses genetic variants, specifically single nucleotide polymorphisms (SNPs), that are associated with an exposure (e.g. expression of a gene) as instrumental variables (IVs), to explore if an exposure causally affects a disease outcome. BN is more flexible in dealing with multiple exposures, but can be computationally expensive when there is a large number of variables in consideration. Badsha and Fu [3] have introduced a machine learning method MRPC that takes forward strengths of both the principle of MR and a constraint-based BN method called the PC algorithm, by looking into undirected and directed relationships from observed data to learn causal structures non-parametrically. However, like other existing network approaches, MRPC suffers from computational intensiveness, and therefore, is not practically applicable to high-dimensional -omics datasets and are only viable for applications to causal interactions between small sets of genes [2].

Hypertension is the single leading cause of premature death worldwide [4, 5]. Genome-wide association studies (GWAS) have found over a thousand SNPs associated with blood pressure (BP) and the risk of hypertension. A large proportion of these SNPs overlap expression quantitative trait loci (eQTLs) in different tissues. At present, it is unclear whether there is a causal influence of gene expression, directly or indirectly, on the risk of hypertension. Indeed, there is a paucity of analytical strategies to examine the causal relationships between gene expression and hypertension.

Kidney is key organ of relevance to BP regulation and the development of hypertension [6–8]. Most post-GWAS functional studies of BP have been restricted to non-renal tissues or kidney tissue with very small sample size [9–11], largely due to a shortage of human kidney collections. We have recently collected age and sex matched kidney genome and transcriptome data for up to 455 kidney samples [12] including healthy and hypertension individuals. This dataset is the largest for the investigation of hypertension to date, which provides a great opportunity of leveraging kidney data to explore the molecular causal network underlying BP, and to help pinpoint novel diagnostic and therapeutic targets for cardiovascular medicine.

Given the complex biology of cardiovascular outcomes and the limitations of current approaches, there is an urgent need for more flexible and comprehensive statistical methods to exploration of their causal mechanisms. Here, we propose a computationally tractable algorithm MRdualPC to infer causal molecular networks based on MRPC [3], which is applied to hypertension data to investigate the upstream causal transcriptomics that influence the disease.

## Methods

### MRdualPC - a faster MRPC algorithm

MRPC, proposed by Badsha and Fu [3], starts with a fully connected undirected network and uses the PC algorithm to test for marginal or conditional independence between pairs of nodes, and to informs which edge(s) to remove. The principle of MR is then applied to orient the edges that remain in the network. Initially, edges are oriented in such a way that they always point from SNPs to molecular phenotypes, because SNP genotypes can be predictive of phenotypic changes but the reverse does not hold. MRPC then looks for v-structures between sets of three nodes and tests for conditional independence to determine the topology of the triplet. A comprehensive description of MRPC is shown in Badsha and Fu [3]. A limitation of this approach lies in its computational inefficiency, particularly in generating the initial undirected networks using the PC algorithm.

In this study, we propose a much faster algorithm named MRdualPC, by utilizing some principles from the recently developed dualPC algorithm [13] to make MRPC more computationally tractable. In dualPC, independence properties of a pair of nodes are tested by conditioning on, simultaneously, an empty set of variables and the full set of (say, *k*) neighbouring variables. It then moves to conditioning on one neighbouring variable and all the remaining *k-1* neighbouring variables, and step by step, moves towards the central order conditional independences until all are tested. In each step, it tests for independence by conditioning on both a variable set and the complementary set, hence the name dualPC. This initial step greatly reduces the time of learning the skeleton of the data structure, as compared to the PC algorithm which tests for independence from lowest to highest order one at a time. Within a fully connected network, the existence of the edge between every pair of nodes will be tested using Fisher’s z-test based on partial correlation. If there is evidence for marginal or conditional independence, the edge will be removed from the network. The R code to apply this initial filtering step in MRdualPC is available at 10.5281/zenodo.8030204. This extra step was taken to quickly and effectively reduce the number of edges in the network before running MRPC. Then the modiSkeleton() function from the MRPC package was adapted to be able to accept a network that has had edges initially removed using dualPC and is available at 10.5281/zenodo.8030204.

### Data collection and pre-processing

Gene expression and eQTL data were combined from five studies (TRANSLATE, RESPOND, ADMIRE, TRANSLATE-T and REPAIR) and processed as described in our previous publication [12]. Kidney samples were included in this study. There were a total of 455 individuals, of which 300 had hypertension and 155 were nonhypertensive. For the 300 hypertension patients, 106 (35.3%) were female and the mean age was 61.7 (range: 20–87). For the 155 control patients, 64 (41.3%) were female and the mean age was 50.0 (range: 18–81). There was a significant difference in age between the hypertension and control groups (t-test, p-value < 2.2e-16). No evidence suggested a significant difference in proportion of females in each group (χ [2] test, p-value = 0.25).

### Workflow of our study

**Fig. 1:**
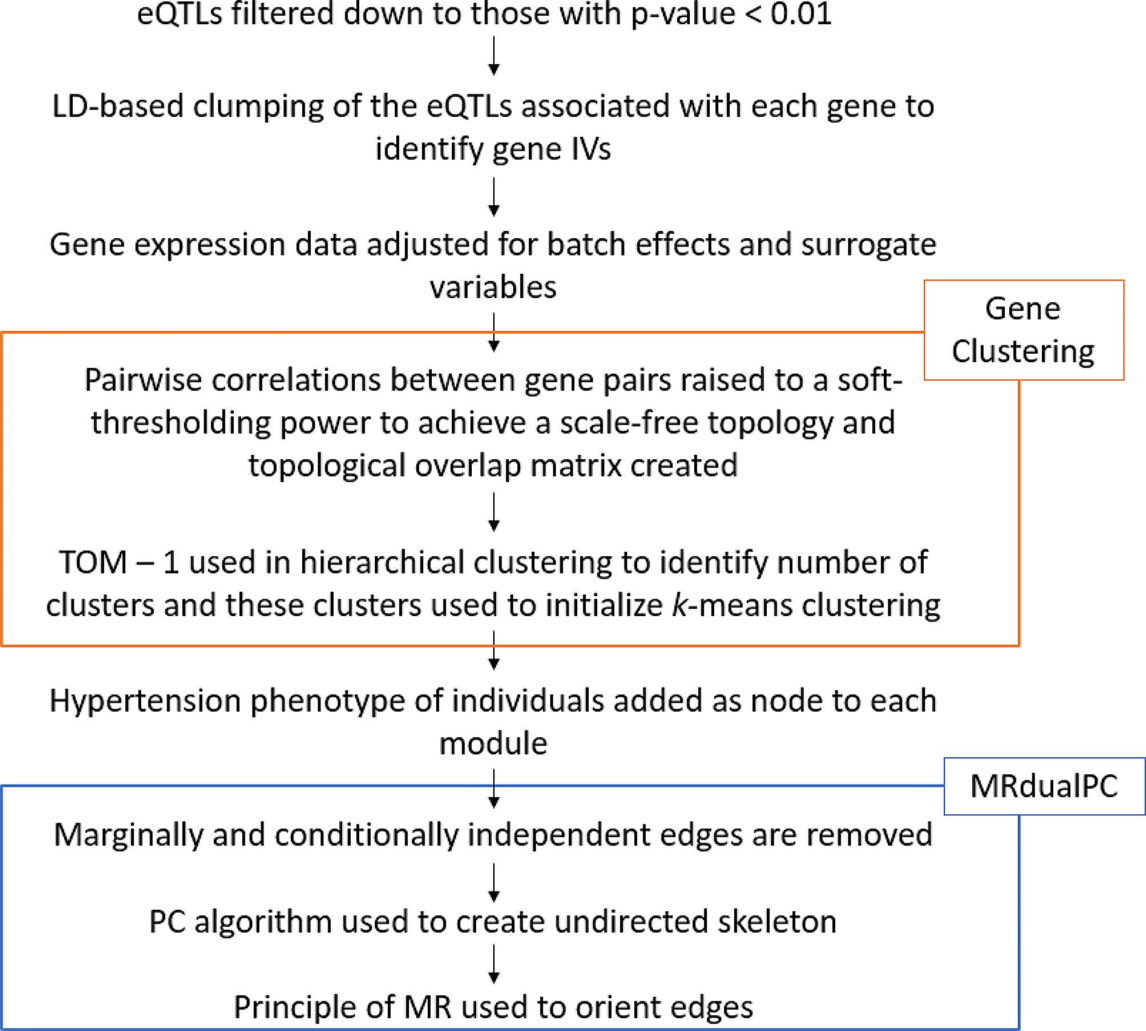
Flowchart of causal network analysis of hypertension using MRdualPC.

The workflow of the study is shown in Fig. 1.

Gene expression-associated SNPs in the summary eQTL data (p-value < 0.01) were retained and mapped from Genome Reference Consortium Human Build 37 (GRCh37) to rsID using the UK Biobank GWAS imputed v3. SNPs in each gene were then clumped based on LD using the TwoSampleMR R package [14]. This is to ensure SNPs selected as IVs are mutually independent. Default settings were used with a clumping window of 10,000 kb and an R [2] cutoff of 0.1. Any genes that had no SNPs mapped to them were removed.

The effect of sex, age and transplant status in the gene expression data was controlled for using the removeBatchEffect() function in the limma package [15], followed by identifying the number of surrogate variables using sva() in the SVA package [16]. The removeBatchEffect function was then applied again to adjust for these surrogate variables. Genes were mapped from Ensembl annotations to gene symbols. For any symbols that mapped to multiple genes, the gene with the highest MAD was kept for downstream analysis, because higher MAD suggests greater variability and therefore, more information [17].

A matrix of correlations between all pairs of genes was created and raised to a soft-thresholding power to achieve a scale-free topology R [2] of 0.85. This was done as there is an assumption that genetic networks operate with as scale free networks, with a small number of highly interconnected hub genes [18]. A scale-free topology R [2] of 0.85 was used as a result of findings from previous studies [19, 20]. As is standard with other gene network approaches, including WGCNA [21], a topological overlap matrix (TOM) was calculated [22], which considers the adjusted correlations between two genes in addition to shared connections with other genes in the network. We converted the TOM to topological overlap dissimilarities to be used with hierarchical clustering. A dynamic tree-cutting algorithm was then adopted to determine initial module assignments of genes (cutreeHybrid, using default parameters except deepSplit of 3, minModuleSize of 40 and mergeCutHeight of 0.3) [23]. The selection of these parameters aims to optimize the number of modules generated. Specifically, we fine-tune them to create modules that are computationally manageable while still capturing significant large-scale interactions. The choice of a minimum module size serves a similar purpose: gene interactions are intricate and multifaceted. Smaller networks of interacting genes could hinder thorough examination and diminish the statistical power for subsequent investigations. An additional k-means clustering step was applied to the topological overlap dissimilarities, initialised using the results of the hierarchical clustering in WGCNA as proposed by Botía et al. [24]. This has been reported to be able to reduce the number of misplaced genes and improving the enrichment of GO pathway terms.

### Causal network analysis of hypertension data

A casual molecular network of hypertension was constructed using MRdualPC for each cluster of genes (also known as a module) and their associated IVs. As described above, edges between variables showing non statistically significant correlation, or in other words, showing no evidence for marginal dependence (p-value > 0.05 in Fisher’s z-test) were removed. No FDR control was applied in this exploratory study. These tests are available as the function filter_module_genes at 10.5281/zenodo.8030204. Next, the network was included as the inputs to the modified ModiSkeleton function from MRPC [3], which performs the PC algorithm to identify the final undirected skeleton. This was run with a p-value threshold (alpha) of 0.05 in conditional independence tests (indepTest = “gaussCItest”). Edges of the network were oriented using the EdgeOrientation function from MRPC [3] using the same arguments as with the modified ModiSkeleton function.

After causal molecular networks were inferred for each gene module, those showing at least a causal pathway to hypertension were identified. The biological pathways associated with the causal ancestors of hypertension were investigated using GO and KEGG 2019 pathway enrichment analysis embedded in the Enrichr web tool [25, 26]. Pathways and GO terms with a p-value < 0.01 were considered statistically significant.

Our recent work identified 885 SNPs associated with at least one blood-pressure (BP) defining trait (systolic BP, diastolic BP or pulse pressure) in previous GWAS studies [27], of which 821 were available in the dataset we used in this study [12]. We used the testGeneOverlap function from the GeneOverlap R package [28] to test if gene expression associated with these GWAS SNPs were over-represented in the causal ancestors of hypertension.

To demonstrate the computational tractability of our adapted MRPC, we processed the data as above, however determined the initial module assignment differently (cutreeHybrid, using default parameters except deepSplit of 4, minModuleSize of 30 and mergeCutHeight of 0.2) in order to generate a larger number of modules at varying sizes. MRPC and our MRdualPC methodology were run on these clusters and time to infer causal networks recorded.

## Results

### Data collection and pre-processing

After filtering summary eQTL data to those with a p-value < 0.01 and mapping to rsID, there remained 1,739,649 unique SNPs that were associated with 17,540 unique genes, with an average of 182.1 SNPs associated with each gene. After linkage disequilibrium (LD)-based clumping, the average number of SNPs associated with each gene was reduced to 6.3. A total of 17,400 genes remained after removal of those not associated with any SNPs.

We identified 31 surrogate variables for adjustment and 17,382 genes after mapping their Ensembl annotations to gene symbols and with the highest median absolute deviation (MAD). The soft threshold power of 11 was identified based on an approximate scale-free topology R [2] to define the adjacency matrix of the gene expression data. Using the topological overlap matrix (TOM), hierarchical clustering identified 98 modules to initialize k-means clustering.

### Causal network analysis of hypertension

MRdualPC was run on each of the 98 modules. Of these modules’ networks, 63 had causal driver genes of hypertension. There were 45 modules (71.4%) that had 5 or more causal gene ancestors of hypertension, 17 modules (30%) with 50 or more causal gene ancestors of hypertension and 6 modules (9.5%) with 100 or more.

**Fig. 2:**
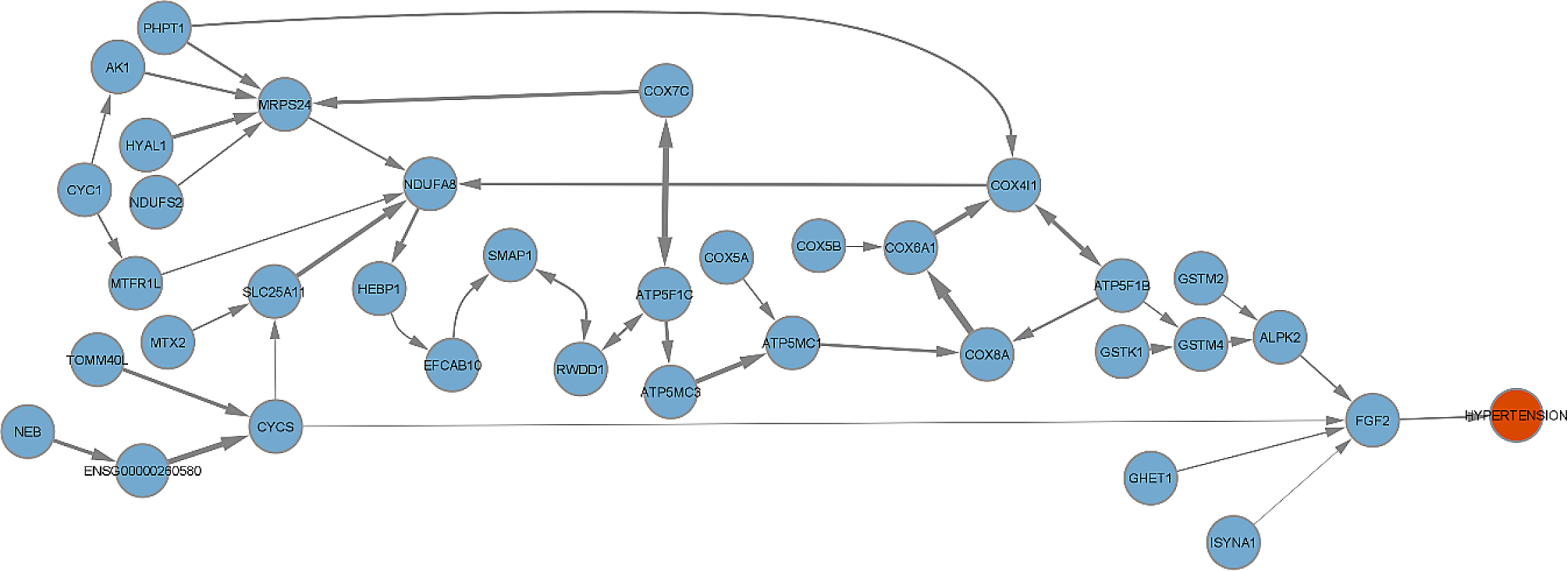
Causal drivers of hypertension identified by MRdualPC in one of 63 modules that had causal ancestor genes of disease. Thickness of edges represent the degree of the pairwise partial correlation conditioned on all other nodes in the network

All modules are available as an interactive shiny application at https://jack-kelly-manchester.shinyapps.io/Causal_molecular_networks_Hypertension/. An example module, shown in Fig. 2, initially contained 173 genes and 1187 IVs, and MRdualPC identified 35 causal ancestor genes that directly and/or indirectly affect hypertension. The genes in this causal network were associated with electron transport chain (gene ontology (GO) biological, p-value = 2.41e-17, overlap = 10/70; wikipathways, p-value = 1.36e-19, overlap = 12/103) and oxidative phosphylation (Kyoto Encyclopedia of Genes and Genomes (KEGG), p-value = 3.553e-20, overlap = 13/133). Included in this network is the gene *ISYNA1*, which we have identified as having a causal association with BP previously [12]. Across all the modules, BP defining trait GWAS associated genes were over-represented in the causal ancestors of hypertension identified using MRdualPC (Odds ratio (OR) = 1.6, p-value = 2.7e-05) with an overlap of 114 genes.

**Fig. 3:**
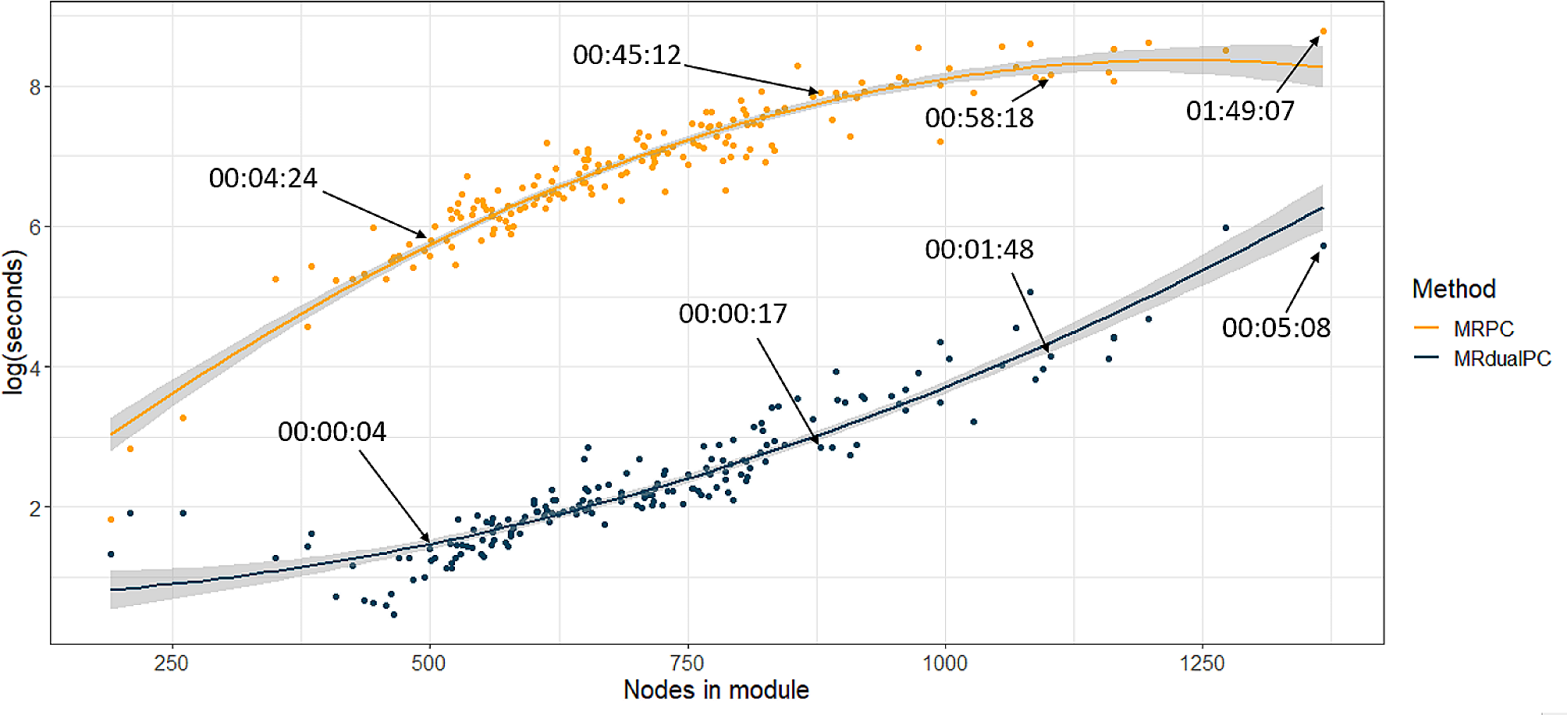
Computation time (in seconds, log-transformed) of MRPC (orange) and MRdualPC (blue) as a function of number of nodes. Each points represent a module used for causal network analysis. Highlighted run-times of the two approaches for four modules are on the original time scale formatted as hours: minutes: seconds

Computational tractability of MRdualPC compared to MRPC is shown in Fig. 3. Data was clustered into 175 modules. On average, MRdualPC was about 100 times faster than MRPC. The largest module which contained 1367 genes and IVs took one hour and 49 min to generate the causal networks using MRPC, and only 5 min when using MRdualPC. Additionally, MRdualPC uses less memory than MRPC making construction of large causal networks more feasible. It is seen that the two fitted curves form a convex. This is mainly because of the high uncertainty in the fitted curves (shaded areas) led by the sparse numbers of modules at each end. The networks generated by MRPC and MRdualPC during this test are available at 10.5281/zenodo.8030204. It is interesting to note that the MRdualPC approach identified results that reflect the above analysis, however MRPC failed to identify any networks with gene ancestors to hypertension.

## Discussion

In this study, we developed MRdualPC, a fast machine learning algorithm for building causal molecular networks. This algorithm was over 100 times faster on average than MRPC in identifying the transcriptomics drivers of hypertension in our study. After clustering, 63 modules appeared to have causal driver genes of hypertension, 17 of which had large causal networks including over 50 genes.

We showed genes in one of the causal networks were associated with the electron transport chain and oxidative phosphorylation, which have previously been associated with hypertension [29]. Within this network, we have previously reported the gene *ISYNA1* as having a causal association with BP [12]. Additionally, we find that the causal ancestor genes of hypertension that were identified by MRdualPC has an over-representation of BP defining trait GWAS associated genes. These shows support for our method as identifying the underlying causal pathways behind hypertension.

MRdualPC has many potential applications beyond gene expression data. The availability of multi-omics data is increasing, and integration of different data types allows for the most complete picture of a disease [30]. For example, MRdualPC could easily be applied to multi-omics data to investigate how gene expression, methylation and other types of omics data interact in their effects on a disease. It can also be used to investigate interactions in omics data in healthy individuals to gain a better understanding of biological mechanisms.

There are some limitations to our approach. Although MRdualPC is much faster than alternative approaches, large gene expression datasets still need clustering for causal inference to be computationally feasible. If integrated omics data are used, the dimensionality of the data will increase greatly and the number of independence tests increase exponentially, and therefore, clustering will become even more important. Clustering multi-omics data is difficult due to high dimensionality and heterogeneity of data and is a developing area of research [31]. Nevertheless, the speed of MRdualPC opens up the possibilities of multi-omics when compared to previous approaches to building causal networks.

In conclusion, we have developed a computationally tractable algorithm based on MRPC [3] to infer large scale causal molecular networks. This approach offers a lot of potential to researchers who have generated or have access to big data. It is also applicable to multi-omics data, a developing area of research that is becoming increasingly important. We apply this approach to investigate the upstream causal transcriptomics that influence hypertension disease and identify known causal pathways including electron transport chain and oxidative phosphorylation and casual genes associated with BP. Our MRdualPC approach represents a step-forward in building causal molecular networks with improved computational cost.

## Data Availability

The datasets used and/or analysed during the current study are available from the corresponding author on reasonable request. All causal networks of hypertension constructed in this study are available as an interactive shiny application at https://jack-kelly-manchester.shinyapps.io/Causal_molecular_networks_Hypertension/. All code of statistical analysis and MRdualPC is available at 10.5281/zenodo.8030204.

## Abbreviations

MR: Mendelian randomization
BN: Bayesian network
SNP: single nucleotide polymorphisms
IV: instrumental variable
GWAS: Genome-wide association studies
BP: blood pressure
eQTL: expression quantitative trait loci
LD: linkage disequilibrium
MAD: median absolute deviation
TOM: topological overlap matrix
WGCNA: weighted gene co-expression network analysis
GO: gene ontology
KEGG: Kyoto Encyclopedia of Genes and Genomes
